# PARG deficiency is neither synthetic lethal with BRCA1 nor PTEN deficiency

**DOI:** 10.1186/s12935-016-0333-2

**Published:** 2016-07-01

**Authors:** Aurélia Noll, Giuditta Illuzzi, Jean-Christophe Amé, Françoise Dantzer, Valérie Schreiber

**Affiliations:** Biotechnology and Cell Signalling, UMR7242 CNRS, Université de Strasbourg, Laboratory of Excellence Medalis, ESBS, 300 Bd Sébastien Brant, CS 10413, 67412 Illkirch, France

**Keywords:** Poly(ADP-ribose), BRCA, Synthetic lethality, Homologous recombination, Cancer

## Abstract

**Background:**

Poly(ADP-ribose) polymerase (PARP) inhibitors have entered the clinics for their promising anticancer effect as adjuvant in chemo- and radiotherapy and as single agent on BRCA-mutated tumours. Poly(ADP-ribose) glycohydrolase (PARG) deficiency was also shown to potentiate the cytotoxicity of genotoxic agents and irradiation. The aim of this study is to investigate the effect of PARG deficiency on BRCA1- and/or PTEN-deficient tumour cells.

**Methods:**

Since no PARG inhibitors are available for in vivo studies, PARG was depleted by siRNA in several cancer cell lines, proficient or deficient for BRCA1 and/or PTEN. The impact on cell survival was evaluated by colony formation assay and short-term viability assays. The effect of simultaneous PARG and BRCA1 depletion on homologous recombination (HR) efficacy was evaluated by immunodetection of RAD51 foci and using an in vivo HR assay.

**Results:**

The BRCA1-deficient cell lines MDA-MB-436, HCC1937 and UWB1.289 showed mild sensitivity to PARG depletion, whereas no sensitivity was observed for the BRCA1-proficient MDA-MB-231, MDA-MB-468, MCF10A and U2OS cell lines. However, the BRCA1-reconstituted UWB1.289 cell lines was similarly sensitive to PARG depletion than the BRCA1-deficient UWB1.289, and the simultaneous depletion of PARG and BRCA1 and/or PTEN in MDA-MB-231 or U2OS cells was not more cytotoxic than depletion of BRCA1 or PTEN only.

**Conclusions:**

Some tumour cells displayed slight sensitivity to PARG deficiency, but this sensitivity could not be correlated to BRCA1- or PTEN-deficiency. Therefore, PARG depletion cannot be considered as a strategy to kill tumours cells mutated in BRCA1 or PTEN.

## Background

Poly(ADP-ribosyl)ation (PARylation) is a post-translational modification of proteins involved in many biological processes, among them the surveillance and maintenance of genome integrity [1, 2]. In response to DNA strand breaks, PARP-1, the founding member of the poly(ADP-ribose) polymerases (PARP) family, immediately and actively transfers single or successive ADP-ribose units from NAD^+^ to acceptor proteins to synthesize poly(ADP-ribose) (PAR) at the damaged site, to signal the lesion and to efficiently and rapidly recruit chromatin modulators and DNA repair factors. PARylation is a transient modification, its kinetics and intensity is tightly controlled through the prompt and efficient degradation of PAR by the glycohydrolase activity of poly(ADP-ribose) glycohydrolase (PARG). PARG exists as multiple isoforms localized to different cellular compartment [3, 4]. In mice, invalidation of all PARG isoforms is embryonic lethal [5], whereas hypomorphic mutant mice or embryonic stem cells are viable but display increased sensitivity to ionizing radiation and alkylating agents [6, 7]. We and others have shown that efficient depletion of all PARG isoforms in cellular models by RNA silencing increased cell death upon genotoxic stress, a consequence of perturbed repair of damaged bases, single and double strand breaks and collapsed replication forks [8–11]. Therefore, both synthesis and degradation of PAR require tight regulation for competent repair [1, 2]. These results define PARG, like PARP-1, as a new candidate target to potentiate chemo- and radiotherapy [8, 12, 13].

That PARP inhibition prejudices efficacy of break repair has been exploited as anticancer strategies to potentiate the cytotoxicity of anticancer drugs [14, 15]. Numerous phase I to III clinical trials based on this approach are in progress [14] (https://www.clinicaltrials.gov/). PARP inhibition has also proved to be exquisitely efficient to kill tumour cells deficient in double strand break repair by homologous recombination (HR), such as cells mutated for the breast cancer early onset (BRCA) genes BRCA1 or BRCA2 [16, 17]. The proposed explanation for this synthetic lethality between PARP and BRCA is that single strand breaks that arise spontaneously in cells are not efficiently repaired upon PARP inhibition, are thus converted during replication to double strand breaks that cannot be repaired in BRCA deficient cells, leading to cell death. The capacity of PARP inhibitor to trap the inhibited PARP-1 onto DNA breaks has been shown to be critical for the cytotoxic effect [18, 19]. The PARP inhibitor olaparib has just been approved for maintenance treatment in advanced ovarian cancers with germline BRCA mutation and several phase III clinical trials are in progress for the treatment of breast and ovarian cancers with BRCA mutations [20].

Whether PARG deficiency could also be cytotoxic to BRCA1/2 deficiency has not been extensively studied. Yet one study showed the increased killing effect of PARG depletion in BRCA2 deficient cells [21]. In the present study, we tackled the hypothesis of synthetic lethality between PARG and BRCA1. Since currently available PARG inhibitors are not satisfying so far, questioned either for their specificity or for their cell permeability [22], we evaluated the impact of PARG depletion by siRNA on cell survival of several breast and ovarian cancer cell lines either proficient or deficient in BRCA1. The phosphatase and TENsin homolog (PTEN) is a tumour suppressor gene regulating the PI3K/AKT signalling pathway [23]. Since synthetic lethality between PARP inhibition and PTEN has also been reported, proposed to result from the impairment of HR caused by PTEN deficiency [24–27], we also examined the effect of PARG depletion on survival of cells endowed with a PTEN deficiency.

## Results

### PARG deficiency is not synthetic lethal with BRCA1 deficiency

**Fig. 1:**
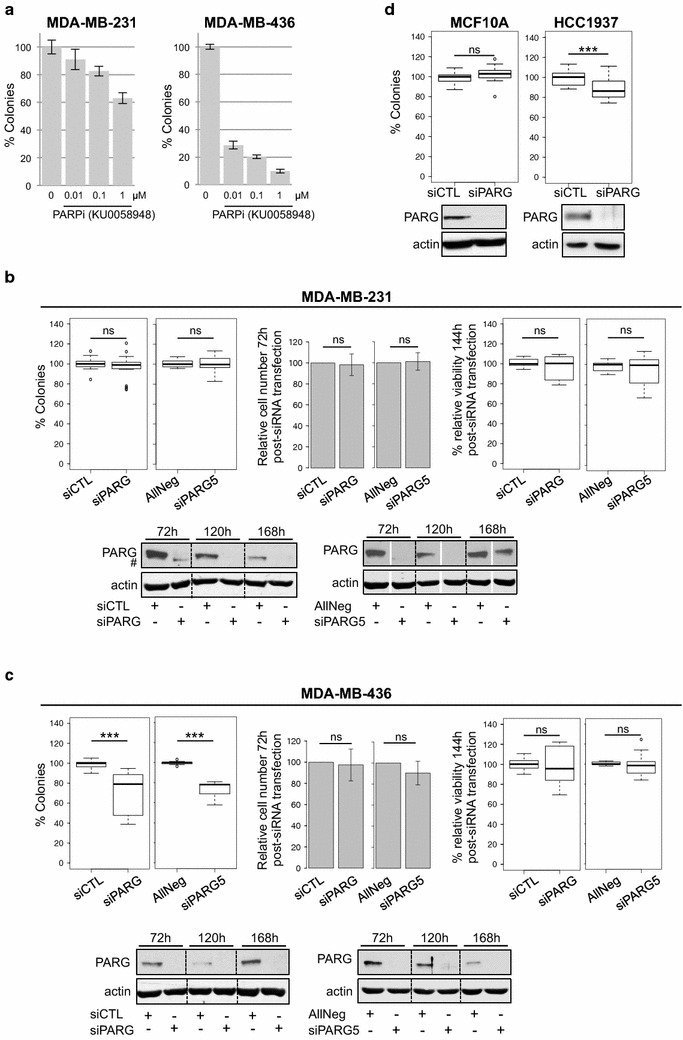
The BRCA1-deficient MDA-MB-436 and HCC1937 cells but not the BRCA-proficient MDA-MB-231 and MCF10A cells are slightly sensitive to PARG depletion. **a** The MDA-MB-436 cell line shows increased sensitivity to PARP inhibition compared to the MDA-MB-231 cell line. Colony formation assays were performed with indicated concentrations of PARP inhibitor KU0058948. Mean values of triplicates (± SD) from one representative of 5 independent experiments are shown. **b** The BRCA1-proficient MDA-MB-231 cell line is not sensitive to PARG depletion. *Left panels* Clonogenic survival of MDA-MB-231 cells transfected with siCTL, siPARG, AllNeg or siPARG5. Results are from 6 (siCTL and siPARG) and 3 (AllNeg and siPARG5) independent experiments. *Middle panels* Percentage of viable cells relative to non-targeting siRNA transfected cells 72 h post-transfection, time point when cells are re-plated for clonogenic or short-term MTS assay. Results show mean values ± SD of 7 (siCTL and siPARG) and 5 (AllNeg and siPARG5) independent experiments.* Right panels* Cell viability measured by MTS assays 144 h post-transfection. Results show the percentage of viability relative to cells transfected with non-targeting siRNA from 3 independent experiments. *Lower panels* PARG depletion was verified by western blot at the time post-siRNA transfection indicated. #: non-specific band. **c** Clonogenic survival (*left panels*), cell counting at 72 h post-siRNA transfection (*middle panels*) and short-term MTS assay at 144 h post-siRNA transfection (*right panels*) were performed in MDA-MB-436 cell line as described in **b**. Number of experiments was 7 (siCTL and siPARG) and 3 (AllNeg and siPARG5) for clonogenic assays, 8 (siCTL and siPARG) and 4 (AllNeg and siPARG5) for cell counting at 72 h and 3 for MTS assay at 144 h post-siRNA transfection. *Lower panels* PARG depletion was verified by western blot at the time post-siRNA transfection indicated. **d** Clonogenic survival of MCF10A (*left panel*) and HCC1937 (*right panel*) cells transfected with siCTL and siPARG. Results are depicted as in **a** from 6 independent experiments*. p*: *** < 0.001; 0.001 < ** < 0.01; 0.01 < * < 0.05; 0.05 < ns, not significant

In order to evaluate whether PARG depletion sensitizes cells mutated in BRCA1 by synthetic lethality, we depleted PARG by siRNA in several breast cancer cell lines either wild type or mutated for BRCA1. We selected the BRCA1-wild type MDA-MB-231 and BRCA1-mutated MDA-MB-436 cell lines and first verified that only the MDA-MB-436 cells were highly sensitive to PARP inhibition in clonogenic assays, as described previously [28–31]. As expected, increasing concentrations of the PARP inhibitor KU-0058948 was far more cytotoxic in MDA-MB-436 than in MDA-MB-231 (Fig. 1a). We next depleted PARG by transient siRNA transfection in these two cell lines and evaluated cell survival by clonogenic assay. Two different siRNAs targeting PARG were used, from different providers, siPARG and siPARG5, with their respective non-targeting controls siCTL, and AllNeg. Efficient knockdown of PARG expression was observed by western blot for both siRNA in both cell lines, for at least 120 h post-transfection, and was even still effective at 168 h in MDA-MB-436 (Fig. 1b, c, lower panels). None of the siRNA targeting PARG had an impact on MDA-MB-231 clonogenic survival (Fig. 1b, left panel). Short-term survival assays similarly revealed no impact on cell number at 72 h post-siRNA transfection or on cell viability 144 h post-transfection, after re-seeding of the cells (Fig. 1b, middle and right panels). This suggests that MDA-MB-231 cells are not particularly sensitive to PARG depletion. Similarly, other BRCA1-wild type cell lines tested, such as the non-tumour MCF10A breast cell line (Fig. 1d), the lung fibroblastic MRC5 cell line (data not shown) and the U2OS osteosarcoma cell line (Fig. 3b) showed no particular sensitivity to PARG depletion.

In contrast, both PARG-targeting siRNA significantly affected clonogenic survival of the BRCA1-deficient MDA-MB-436 cell line (Fig. 1c, left panel). PARG depletion had however no significant impact on cell number at 72 h post-siRNA transfection or on cell viability 144 h post-transfection (Fig. 1c, middle and right panels, respectively). We concluded that clonogenic assay is more sensitive to uncover an eventual impact of PARG depletion on cell survival that is rather weak, probably because in this technique, cells have to recover from plating in discriminating conditions caused by their extreme dilution. SiPARG and siPARG5 impacted clonogenic survival of MDA-MB-436 cell line to similar extent, validating their specificity. The fact that another BRCA1-impaired cell line, HCC1937, also displayed mild sensitivity to siPARG (Fig. 1d) could be in favour of a possible synthetic lethality between PARG depletion and BRCA1 deficiency.

**Fig. 2:**
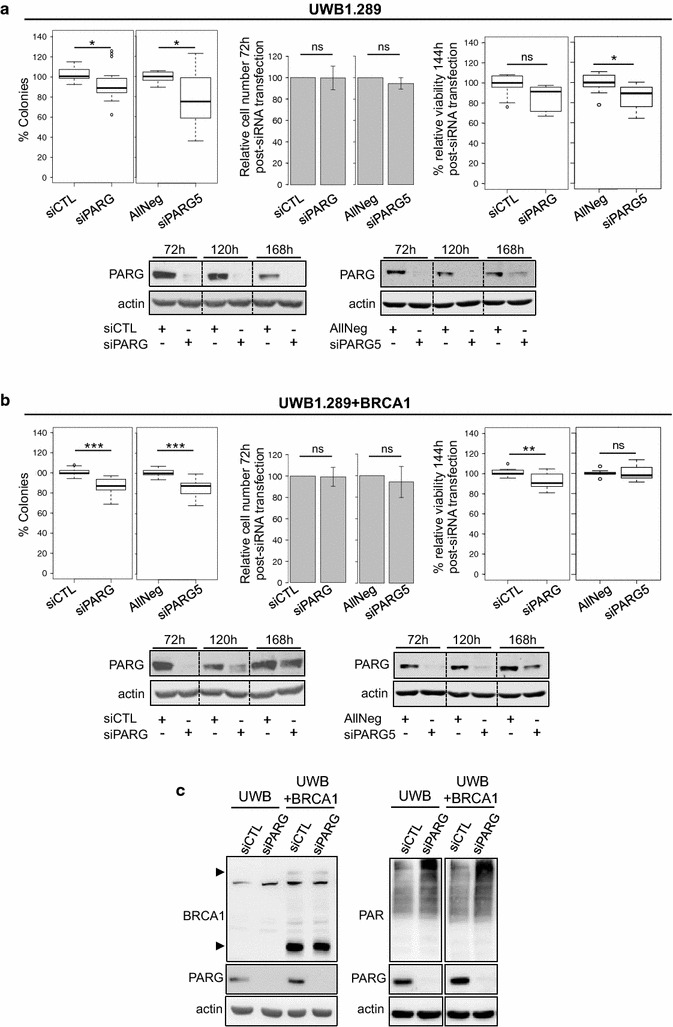
The BRCA1-mutated UWB1.289 cell line is not more sensitive to PARG depletion than the BRCA1-reconstituted UWB1.289 + BRCA1 cell line. **a** *Left panels* Clonogenic survival of UWB1.289 cells transfected with siCTL, siPARG, AllNeg or siPARG5. Results are depicted as* box plots* showing distribution of data from 7 (siCTL and siPARG) and 4 (AllNeg and siPARG5) independent experiments. *Middle panels* Percentage of viable cells relative to non-targeting siRNA transfected cells 72 h post-siRNA transfection, time point when cells are re-plated for clonogenic or short-term MTS assay. Results show mean values ± SD of 11 (siCTL and siPARG) and 4 (AllNeg and siPARG5) independent experiments. *Right panels* Cell viability measured by MTS viability assays 144 h post-transfection. Results show the percentage of viability relative to cells transfected with non-targeting siRNA from 3 independent experiments. *Lower panels* PARG depletion was verified by western blot at the time post-siRNA transfection indicated. **b** Clonogenic survival (*left panels*), relative cell number at 72 h post-siRNA transfection (*middle panels*) and short-term MTS assay at 144 h post-siRNA transfection (*right panels*) were performed in UWB1.289 + BRCA1 cell line exactly as described in **a**. Results are depicted as *box plots* showing distribution of data from 7 (siCTL and siPARG) and 4 (AllNeg and siPARG5) for clonogenic assays. Number of experiments was 11 (siCTL and siPARG) and 4 (AllNeg and siPARG5) for cell counting at 72 h and 3 for MTS assay at 144 h post-siRNA transfection. *Lower panels* PARG depletion was verified by western blot at the time post-siRNA transfection indicated. **c**. Spontaneous PAR accumulation is a consequence of efficient PARG depletion in UWB1.289 (UWB) and UWB1.289 + BRCA1 (UWB + BRCA1) cells. PAR, BRCA1, PARG and actin levels were analysed by western blot using the indicated antibodies. BRCA1 specific signal is indicated by *arrowheads. p*: *** < 0.001; 0.001 < ** < 0.01; 0.01 < * < 0.05; 0.05 < ns, not significant

To examine this hypothesis, the sensitivity to PARG depletion of another cancer cell line genetically deficient for BRCA1 was compared to that of the corresponding BRCA1-reconstituted cell line. We selected the BRCA1-deficient ovarian cancer cell line UWB1.289 and the corresponding UWB1.289 + BRCA1 that expresses functional human BRCA1 (Fig. 2a, b) [32]. PARG depletion was controlled by western blot, and revealed to be very efficient for both siRNA targeting PARG at 72 h post-transfection, until at least 120 h post-transfection. Efficient PARG depletion was also supported by the strong PAR increase, spontaneously accumulating at similar levels in both PARG-depleted cell lines (Fig. 2c). Clonogenic assays revealed that both PARG-targeting siRNA slightly but significantly decreased cell survival of both cell lines, regardless of their BRCA1 status (Fig. 2a, b, left panels). Here again, short-term assays were less informative, with no significant difference observed at 72 h post-transfection for all siRNA and only a slight decrease of viability observed at 144 h, significant only for siPARG5 in UWB1.289 and siPARG in UWB1.289 + BRCA1 cells (Fig. 2a, b, middle and left panels). Therefore, considering results obtained with the more robust clonogenic assay, synthetic lethality between PARG depletion and BRCA1 deficiency was not supported in the UWB1.289 cells.

**Fig. 3:**
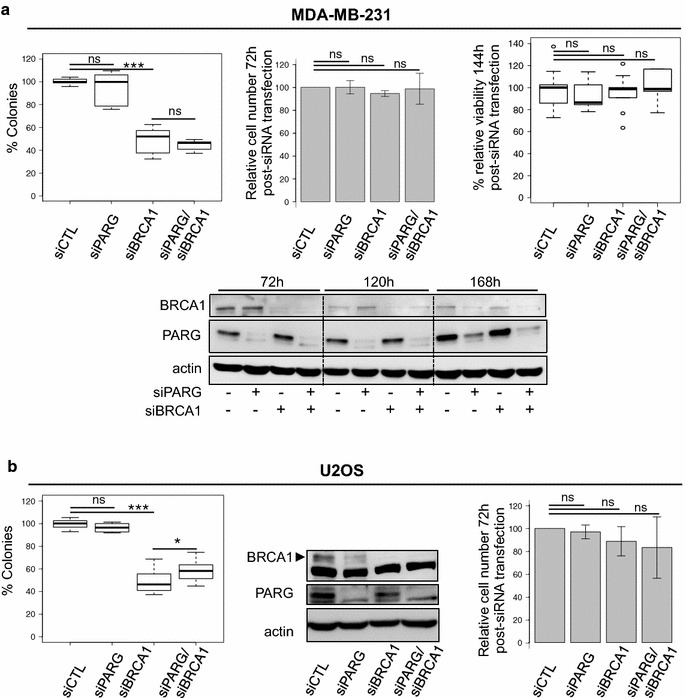
Co-depletion of PARG does not potentiate cytotoxicity by BRCA1 depletion. **a** Clonogenic survival (*left panel*) of MDA-MB-231 cells after single or combined siRNA-mediated depletion of BRCA1 and PARG. Results are depicted as *box plots* showing distribution of data from 4 individual experiments. *Middle panel* Percentage of viable cells relative to siCTL-transfected cells 72 h post-transfection, time point when cells are re-plated for clonogenic or short-term MTS assay. Results show mean values ± SD of 7 independent experiments. *Right panel* Cell viability measured by MTS viability assays 144 h post-transfection. Results show the percentage of viability relative to cells transfected with siCTL from 3 independent experiments. *Lower panel* PARG and BRCA1 depletions were verified by western blot at the times indicated. **b** Clonogenic survival (*left panel*) of U2OS cells after single or combined siRNA-mediated depletion of BRCA1 and PARG. Results are depicted as *box plots* showing distribution of data from 4 individual experiments. Percentage of viable cells relative to siCTL-transfected cells 72 h post-transfection (*right panel*). Results show mean values ± SD of 5 independent experiments. PARG and BRCA1 depletions at 72 h were verified by western blot (*middle panel*). BRCA1 specific signal is indicated by *arrowhead*. *p*: *** < 0.001; 0.001 < ** < 0.01; 0.01 < * < 0.05; 0.05 < ns, not significant

To clarify our observations, we simultaneously depleted PARG and BRCA1 by siRNA in the BRCA1-proficient MDA-MB-231 cells (Fig. 3a). Western blot analyses with anti-BRCA1 and anti-PARG antibodies confirmed the efficient silencing of both genes (Fig. 3a). Whereas the single BRCA1 depletion shows cytotoxicity, as previously reported [33], decreasing the clonogenic survival to 57 ± 5 %, the simultaneous depletion of PARG did not significantly enhance cell mortality (Fig. 3a, left panel). Of note even the siBRCA1, that dramatically reduces clonogenic survival when used alone or in combination with siPARG, had no effect on short-term survival, at 72 h or 144 h post-transfection (Fig. 3a, middle and right panels). This supports that cell plating at very low density is critical to uncover an altered survival capacity. Comparable results were observed by clonogenic assay when the siRNA-mediated depletion of BRCA1 and/or PARG was performed in the BRCA1-proficient U2OS osteosarcoma cell line, with even a slight increase in clonogenic survival of cells simultaneously depleted for PARG and BRCA1 compared to cells depleted for BRCA1 only (Fig. 3b). Taken together, these results further support the conclusion of a lack of synthetic lethality between PARG and BRCA1.

**Fig. 4:**
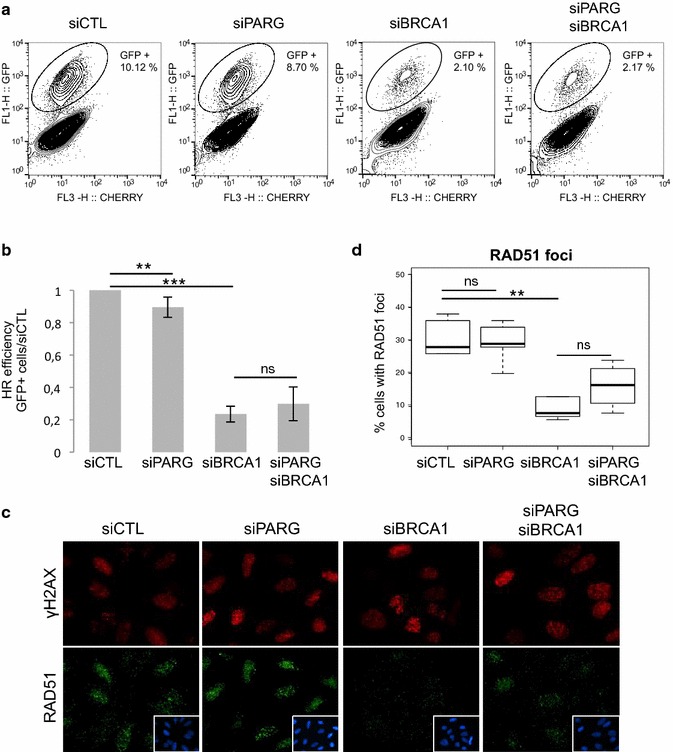
The HR defect caused by BRCA1-depletion is not further affected by PARG depletion. **a** and **b** The frequency of HR-mediated repair events was analysed by flow cytometry in U2OS-DR-GFP-mCherry-I-SceI-GR cells after transfection with the indicated siRNA and induction of DSB formation by incubation with TA for 48 h. The percentage of Cherry- and GFP-positive cells is indicated. In **b**, the values correspond to the ratio of GFP-positive cells relative to cells transfected with scrambled siRNA (siCTL), as illustrated in **a**, and represent the mean ± SD of 7 independent experiments. **c** and **d** Defect of etoposide-induced RAD51 foci formation in BRCA1-depleted cells is not further affected by PARG depletion. Cells transfected with the indicated siRNA were incubated with 10 µM etoposide for 1 h, released into fresh medium for 2 h and processed for immunofluorescence using anti-RAD51 and anti-γH2AX antibodies. Representative immunofluorescence image are shown in **c**. In **d**, the *box plot graph* depicts the percentage of cells displaying RAD51 foci (more than 10 RAD51 foci per cell) from 5 independent experiments scoring >200 nuclei for each condition. *p*: 0.01 < * < 0.05; 0.05 < ns, not significant

To verify that the siRNA-mediated depletion of BRCA1 was functionally effective, we examined its impact on HR-efficiency. To this end, we utilized the in vivo HR assay based on the HR-inducible U2OS-DR-GFP cell line, containing a stably integrated DR-GFP reporter and expressing the mCherry-*I*-*Sce*I-GR fusion protein [34]. Upon binding to triamcinolone acetonide (TA), the mCherry-I-SceI-GR translocates from cytoplasm to nucleus to cleave an I-SceI site present in one of the two integrated mutated GFP-sequences, generating a DSB. Reconstitution of GFP after *I*-SceI-dependent HR was monitored by flow cytometry in cells transfected with siRNA targeting PARG, BRCA1 or both (Fig. 4a, b). In agreement with our previous observations [11], siPARG alone slightly reduced HR compared to scramble siCTL (0.89 ± 0.06 %). In contrast, BRCA1 depletion dramatically reduced HR efficiency (0.23 ± 0.05 %), as expected. In the context of simultaneous PARG and BRCA1 depletion, HR was similarly impaired (0.29 ± 0.11 %) than in the context of the sole BRCA1 depletion, suggesting that BRCA1 is efficiently silenced, affecting HR regardless of the presence of PARG.

To further examine the consequence of PARG and/or BRCA1 siRNA-mediated depletion on HR efficiency, we examined by immunofluorescence the appearance of RAD51 foci in U2OS cells treated with the DSB inducer, topoisomerase II inhibitor etoposide. Cells were treated with 10 µM etoposide for 1 h and released in complete fresh medium for additional 2 h (Fig. 4c, d), time chosen from kinetic experiment as the best moment to detect RAD51 foci in these conditions of treatment (data not shown). The presence of DSB was revealed by co-staining for γH2AX (Fig. 4c). We have reported previously that RAD51 foci formation was impaired in PARG-depleted cells in conditions of massive PAR production caused by prolonged replicative stress known to generate DSB [11]. Etoposide is a good inducer of DSB but a poor trigger of PAR synthesis [35]. In agreement, only limited PAR synthesis was observed in etoposide-treated PARG-depleted cells (data not shown) and no significant difference of RAD51 foci formation was detected between siCTL and siPARG cells (Fig. 4c, d). BRCA1 depletion dramatically impaired the formation of RAD51 foci, as expected, with only 9.4 ± 3.4 % cells with RAD51 foci compared to 30.8 ± 5.8 % in siCTL cells. In the context of simultaneous PARG and BRCA1 depletion, more cells showed RAD51 foci (16.2 ± 6.85 %) but this difference was not statistically significant compared to the condition of the sole BRCA1 depletion (p = 0.37). This result is in complete accordance with the results of the in vivo HR analysis described above, demonstrating that BRCA1 deficiency affects HR and that simultaneous PARG depletion has no impact on this HR defect.

### PARG deficiency is not synthetic lethal with PTEN deficiency

In addition of being deficient in BRCA1, the MDA-MB-436 and HCC1937 cells that display a slight sensitivity to PARG depletion (Fig. 1b, d), are also deficient in PTEN, a tumour suppressor gene regulating the PI3K/AKT signalling pathway [23]. Synthetic lethality between PARP inhibition and PTEN has been described for several cancer cell lines and proposed to result from the impairment of HR caused by PTEN deficiency [24–27]. In glioblastoma cell lines, PARP inhibition exacerbated the PTEN-dependent down-regulation of RAD51 transcriptional expression, thus impairing HR [25, 36]. However, other findings have not supported these results, showing no impact on RAD51 expression and foci formation by PTEN deficiency, and only mild sensitivity of PTEN-deficient cells to PARP inhibition in primary prostate cancer cell lines and xenografts [37]. Furthermore, a recent study showed that the concurrent loss of PTEN and BRCA1 rather counteracts the HR-repair deficiency, conferring PARP inhibitor resistance [38].

**Fig. 5:**
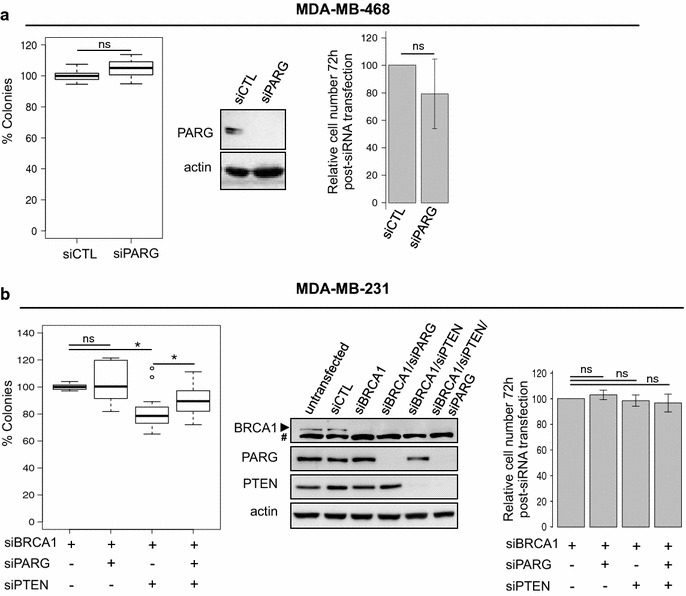
PARG deficiency is not synthetic lethal with PTEN deficiency. **a** The PTEN-mutated MDA-MB-468 cell line is not sensitive to PARG depletion. Clonogenic survival (*left panel*) of MDA-MB-468 cells after siCTL and siPARG transfection. Results are depicted as *box plots* showing distribution of data from 5 individual experiments. Relative cell number 72 h post-siRNA transfection (*right panel*) is counted from 5 individual experiments. PARG depletion was verified by western blot (*middle panel*). **b** Clonogenic survival (*left panel*) of MDA-MB-231 cells after single or combined siRNA-mediated depletion of BRCA1, PARG and PTEN. Results are depicted as *box plots* showing distribution of data from 5 independent experiments. Percentage of viable cells relative to siCTL-transfected cells 72 h post-transfection (*right panel*) is counted from 5 individual experiments. BRCA1, PARG and PTEN siRNA-mediated depletions compared to untransfected or siCTL transfected cells were verified by western blot (*middle panel*). The *arrow* points to BRCA1 signal above a non-specific band (#). *p*: 0.01 < * < 0.05; 0.05 < ns, not significant

In order to challenge the hypothesis of synthetic lethality between PARG and PTEN, we depleted PARG by siRNA in the PTEN-mutated but BRCA1-wild type MDA-MB-468 cell line and evaluated clonogenic survival. PARG depletion had no impact on the viable cell number at 72 h after siRNA transfection of MDA-MB-468 cells, and did not affect the clonogenic survival of MDA-MB-468 cells, inferring that PARG-deficiency is not synthetic lethal with a PTEN mutation (Fig. 5a). In order to assess whether the simultaneous deficiency in BRCA1 and PTEN was necessary to confer sensitivity to PARG depletion, we performed the triple depletion of PARG, BRCA1 and PTEN by siRNA in MDA-MB-231 cells and compared it to the single BRCA1 depletion, and to BRCA1/PARG and BRCA1/PTEN double depletions (Fig. 5b). Cells transfected with siBRCA1 were used here as the reference, since single BRCA1 depletion already strongly affects clonogenic survival (Fig. 3 and [33]). Cell number, at the time of plating for clonogenic assay didn’t vary from that of siBRCA1-transfected cells for any combination of siRNA used (Fig. 5b, right panel). Clonogenic assay revealed that PARG silencing was not cytotoxic to BRCA1/PTEN-depleted cells and rather slightly increased their clonogenic survival (Fig. 5b). Taken together, these results demonstrate that PARG silencing is neither synthetic lethal with BRCA1 nor with PTEN deficiency.

## Discussion

Using cancer cell lines genetically deficient in BRCA1 and/or PTEN and siRNA-mediated depletions, our study shows that PARG deficiency is neither synthetic lethal with BRCA1 nor with PTEN deficiency. The fact that some cells (MDA-MB-436, HCC1937, UWB1.299) display decreased clonogenic survival to PARG depletion cannot be attributed to their BRCA1 and PTEN deficiency, since the UWB1.299 cell line complemented with BRCA1 (UWB1.299 + BRCA1) displayed the same sensitivity to PARG depletion than the parental BRCA1-deficient UWB1.299 cell line. Moreover, we showed that PARG depletion does not sensitize cells simultaneously depleted of BRCA1 or PTEN, or both. Although we cannot exclude that PARG knockdown is incomplete and that residual functional PARG could be still sufficient to prevent cytotoxicity in BRCA1- and/or PTEN-deficient cells, the accumulation of PAR observed in cells transfected with PARG siRNA supports efficient PARG depletion. Specific and cell permeable PARG inhibitors are eagerly expected to confirm these findings.

Whereas PARG depletion is not synthetic lethal with BRCA1 deficiency, it was shown to be cytotoxic to BRCA2-deficient cells [21]. A possible explanation for this different sensitivity between BRCA1 and BRCA2-deficient cells could be that BRCA1 acts early in the HR process, at the level of the repair pathway choice between HR and Non Homologous End Joining (NHEJ), whereas BRCA2 acts at later step of the HR process [39]. In the absence of BRCA1, HR is not initiated and NHEJ can operate to repair the DSB. When HR is engaged but halted by the absence of BRCA2, NHEJ cannot take over to finish the repair. In light with this, it was shown that one of the mechanism of acquired resistance of HR-deficient cells towards PARP inhibitors was the mutation of NHEJ factors, such as 53BP1 or REV7 [40, 41]. Inactivation of NHEJ leads to the partial restoration of homology-directed repair, but this is possible only in BRCA1-deficient, but not in BRCA2-deficient cells. Whether PARG depletion similarly allows bypass of BRCA1 but not BRCA2 function needs further investigation. This is however a tempting hypothesis that could explain why PARG deficiency is cytotoxic to BRCA2-depleted cells [21], but not BRCA1-depleted cells (this work).

Nevertheless, the fact that some cells display certain sensitivity to PARG depletion supports the idea that targeting PARG expression or activity could be considered as an anticancer strategy. PARG depletion was shown to affect cell proliferation of LoVo colon cancer cells line [42]. Several examples however showed that in the absence of exogenous genotoxic stress, PARG depletion did not significantly alter cell proliferation or survival, as observed in MCF7 cells [21, 43], HeLa cells [8] and MDA-MB-231 cells [44]. In contrast, PARG-depletion was shown to sensitize tumour cells to genotoxic insult caused by ionizing radiations and mild but not severe concentrations of alkylating agents or hydrogen peroxide [8, 10, 13, 43]. But even the radiosensitization by PARG deficiency should not be generalized, since some lung tumour cell lines showed no potentialization of radiotoxicity by PARG depletion [45]. The next challenging question will be to determine the molecular signature that modulates the sensitivity or resistance to PARG invalidation.

## Conclusions

Our study shows that although some tumour cells display slight sensitivity to PARG deficiency, this sensitivity cannot be correlated to BRCA1- or PTEN- deficiency. Therefore, PARG depletion cannot be considered as a strategy to kill tumour cells mutated in BRCA1 or PTEN.

## Methods

### Cell lines

Except when indicated, all cell lines were obtained from the American Type Culture Collection (ATCC). MDA-MB-231 and MDA-MB-436 breast cancer cells were cultivated in RPMI 1640, 10 % foetal bovine serum (FBS, Pan Biotech) and 1 % gentamycin (Invitrogen). MDA-MB-436 have a 5396 + 1G >A mutation in the splice donor site of BRCA1 exon 20 [46] and do not express PTEN [23]. The BRCA1 wild type but PTEN-null MDA-MB-468 basal-like breast cancer cells were cultivated in DMEM/F12 HAM (Sigma) supplemented with 2.5 mM l-Glutamine (Invitrogen), 10 % FBS and 1 % gentamycin. The non-tumourigenic epithelial MCF10A breast cell line expresses wild type BRCA1 and PTEN [38] and was cultivated in DMEM/F12 HAM, 5 % horse serum, 0.01 mg/ml insulin, 20 ng/ml human epidermal growth factor, 500 ng/ml hydrocortisone, 100 ng/ml cholera toxin and 1 % gentamycin. The osteosarcoma U2OS cell line was cultivated in DMEM, 10 % FBS and 1 % gentamycin. The PTEN-null and BRCA1-mutated HCC1937 cell line that carries mutated *BRCA1* (5382insC) and are homozygous for PTEN deletion [23, 47] was obtained from J. Chen (MD Anderson Cancer Center, Houston, TX) and cultivated in RPMI 1640, 10 % FBS, 1 % gentamycin. The human ovarian cancer cell line UWB1.289 is BRCA1-defective (2594delC mutation and deletion of the wild type allele) [32]. The UWB1.289 + BRCA1 cell line stably expresses full length human BRCA1 [32]. Both cell lines were cultivated in 50 % RPMI-1640, 50 % mammary epithelial growth medium (Lonza), 3 % FBS and 200 µg/ml G-418 (for UWB1.289 + BRCA1).

### Clonogenic survival assays

Cells were seeded in 6 cm plates and transfected the day after with the different siRNA. Two different siRNAs targeting PARG were used, from two different providers: the ON-TARGETplus SMARTpool from Dharmacon, termed siPARG and the Hs-PARG5-Flexitube from Qiagen, termed siPARG5. Their respective non-targeting controls were: ON-TARGETplus Non-targeting pool from Dharmacon, termed siCTL, and All Stars negative control from Qiagen, termed AllNeg. SiRNA targeting BRCA1 (siBRCA1) and PTEN (siPTEN) were ON-TARGETplus SMART pool from Dharmacon. Depending on the cell line, either JetPRIME (Polyplus) or INTERFERin (Polyplus) transfecting agents were used, with a maximum of 50 nM or 15 nM siRNA, respectively. For co-transfections, equivalent amounts of the different siRNA were mixed and when necessary, completed with siCTL to reach the maximum siRNA concentration. Cells were trypsinized 72 h post-transfection, seeded in triplicates on Petri dishes (10 or 6-cm) and grown for 10–14 days. The optimal number of cells seeded and the duration of culture were established for each cell line. For survival assays performed in the presence of the PARP inhibitor, cells were seeded in complete medium supplemented with the PARP inhibitor Ku-0058948 [17] at the indicated concentration. Colonies were fixed in 3.7 % formaldehyde and stained with 0.1 % crystal violet. Clones with more than 50 cells were counted on scanned images using Image J, using the maxima intensity detection and substraction of background. Determination of minimum clone size was performed under light microscopy. Results are represented as the percentage of survival colonies within each set of experimental data relative to the respective non-targeting control siRNA.

### Short term viability assays

Seventy-two hours post-siRNA transfection, cells were trypsinized and total viable cells were counted. Relative cell number was calculated as the percentage of viable cells relative to the number of cells for the respective non-targeting control siRNA (siCTL or AllNeg), for at least 3 and up to 11 experiments (indicated in figure legend). Short term viability assays was evaluated by a 3-(4,5- dimethylthiazol-2-yl)-5-(3-carboxymethoxyphenyl)-2-(4-sulfophenyl)-2*H*- tetrazolium (MTS) assay (CellTiter 96 Aqueous One Solution Cell Proliferation Assay (Promega) according to the manufacturer’s instructions. After cell counting 72 h post-siRNA transfection, cells were plated in triplicate into 96-well tissue culture plates and cultivated for up to 96 h. The optimal number of cells to be plated was determined for each cell line. Every 24 h, 20 μl of CellTiter 96^®^ AQueous One Solution Cell Proliferation Reagent (Promega) were added into each well of one plate with a multichannel pipette, and after 1–3 h of incubation at 37 °C in a humidified, 5 % CO_2_ atmosphere, the reaction was stopped with 50 μl of a 10 % SDS solution. Absorbance was measured at 490 nm using a 96-well plate reader (Biochrom Asys UVM340). The 144 h post-siRNA transfection time point was selected as representative of short-term MTS viability, defined as the percentage of cell viability relative to the respective non-targeting control siRNA.

### Statistical analyses

Statistical analyses were carried out using R statistical packages. For clonogenic assays, short-term MTS viability assays and RAD51 foci analyses, data are represented as box plot graph where the lower and upper hinges represent the 25th and 75th percentile respectively. The middle horizontal line represents the median or 50th percentile. Whiskers are drawn to the lower and upper adjacent values. Far out values are represented by small “o”. Significance tests, such as Anova and TukeyHSD (honest significant difference, for multiple comparison) were performed in R using the dataset used to draw the box plot. For cell counting at 72 h post-transfection and HR-assays, data are represented as bar plot graphs with standard deviation (SD) and significance evaluated using Student *t* test. The number of independent experiments is indicated in figure legend. For significance codes *p*: 0 <‘***’ <0.001 <‘**’ <0.01 <‘*’ <0.05 <ns (not significant).

### Western blot

Cells remaining after the seeding for clonogenic assays were pelleted by centrifugation, lysed in 20 mM Tris HCl pH 7.5, 400 mM NaCl, 5 mM DTT, 20 % glycerol, 0.1 % NP40, 1 mM Pefabloc, Protease Inhibitory Cocktail (Roche), phosSTOP (Roche), 100 nM Ku-0058948, 1 µM ADP-HPD (Trevigen) and analysed by western blot as previously described [11]. Antibodies used were rabbit anti-PAR (1/1000, 4336-BPC-100, Trevigen), anti-PARG (1/2000, [8]), anti-actin (1/500, A2066, Sigma), anti-PTEN (1/2000, ab154812, Abcam) and anti-BRCA1 (1/5000, 07-434, Millipore) antibodies. Secondary antibodies were either an Alexa Fluor 680 goat anti-rabbit (1/30,000, Invitrogen) or a peroxidase-coupled goat anti rabbit (1/50,000, Invitrogen), revealed either with Odyssey Infrared Imaging System (Li-Cor, Bioscience) or by chemiluminescence and autoradiography.

### HR-assay

HR was performed as described in Illuzzi et al. [11], using U2OS cells containing the HR reporter DR-GFP and the inducible mCherry-I-SceI-GR (U2OS-DR-GFP- mCherry-I-SceI-GR). Cells were transfected with the respective siRNA twice, with interval of 48 h, treated for 2 days with 100 ng/ml of triamcinolone acetonide (TA, Sigma) to induce nuclear translocation of the mCherry-I-SceI-GR before evaluation of the GFP-positive cells out of the mCherry-positive cells by flow cytrometry (FACSCalibur and Cell Quest software, Becton–Dickinson).

### Immunofluorescence

Cells grown on glass coverslips were left untreated or treated with etoposide at 10 µM for 1 h, washed twice with PBS and incubated in complete medium for 2 h. Immunodetection of RAD51 and γH2AX was performed as described in Illuzzi et al. [11], using mouse monoclonal anti γH2AX (Ser139) (IgG1, 1/2000, 05-636, Upstate) antibody and rabbit polyclonal anti-Rad51 (1/1000, H-93, Santa Cruz Biotechnology) antibody. Secondary antibodies were an Alexa Fluor 568 goat anti-mouse IgG and an Alexa Fluor 488 goat anti-rabbit IgG (1/2000, Molecular Probes, Invitrogen). DNA was counterstained with Dapi.

## Abbreviations

BRCA1/2: breast cancer ½
DSB: double strand break
HR: homologous recombination
PAR: poly(ADP-ribose)
PARG: poly(ADP-ribose) glycohydrolase
PARP: poly(ADP-ribose) polymerase
PTEN: phosphatase and tensin homolog
